# An Evaluation of Clinician and Informatician Perspectives on Medication Harm Risk Prediction Tools for Use in a Tertiary Hospital Setting

**DOI:** 10.1111/jep.70592

**Published:** 2026-09-13

**Authors:** Jonathan Yong Jie Lam, Michael Barras, Ian A. Scott, Ahmad Abdel‐Hafez, Nazanin Falconer

**Affiliations:** ^1^ School of Pharmacy and Pharmaceutical Sciences The University of Queensland Brisbane Queensland Australia; ^2^ Department of Pharmacy Princess Alexandra Hospital Brisbane Queensland Australia; ^3^ Centre for Health Services Research The University of Queensland Brisbane Queensland Australia; ^4^ Metro South Digital Health and Informatics Division Princess Alexandra Hospital Brisbane Queensland Australia; ^5^ College of Computing and Information Technology University of Doha for Science and Technology Doha Qatar

**Keywords:** medical informatics, patient safety, qualitative research

## Abstract

**Introduction:**

Risk prediction tools (RPTs) use clinical data to estimate the likelihood of adverse events such as medication harm and institute preventive strategies. However, their adoption in hospitals can be limited by implementation challenges, workflow integration barriers, and technical constraints.

**Objectives:**

This study aimed to determine clinicians' and informaticians' perspectives on the requirements, factors influencing adoption of digital RPTs for medication harm in hospitals, and to identify effective implementation strategies.

**Methods:**

Semi‐structured interviews were conducted with clinicians and informaticians in three Australian digital hospitals within the Metro South Hospital and Health Service in 2025. Themes were inductively identified and deductively mapped to the Integrated Promoting Action on Research Implementation in Health Services (i‐PARIHS) framework.

**Results:**

Twenty‐two interviews were conducted with six doctors, eleven pharmacists, and five informaticians. Five themes were identified, reflecting key influences on the implementation of digital RPTs in hospitals, including clinician trust, perceived utility, and organisational readiness. Desired features such as automation, feedback functionality, and workflow compatibility were seen as essential to adoption. While enablers included comprehensive feedback mechanisms and adequate resource planning, barriers included poor integration with existing systems and uncertainty around tool reliability.

**Conclusion:**

Stakeholders recognised the value of digital RPTs in supporting medication safety. Understanding their perceptions and experiences is necessary for addressing barriers and enhancing tool acceptability. This study highlighted desirable tool features and organisational factors that influence successful implementation. These findings can inform strategies for effective implementation, adoption, and sustained use of RPTs in hospital settings.

## 1 Introduction

Medication harm is a significant healthcare challenge arising from prescribing, dispensing, and administration errors, which can lead to inappropriate medication use, resulting in prolonged hospital stays, increased costs, and poorer patient outcomes [1]. Traditional patient safety approaches, such as incident reporting and root cause analysis, are reactive [2]. Clinicians need more effective and proactive methods to identify patients most vulnerable to medication harm and improve medication safety [3].

Risk prediction tools (RPTs) are analytical models that use clinical and demographic predictors such as age, comorbidities, laboratory results to predict the probability of experiencing a health‐related event or outcome [4]. The development of RPTs is driven by the need to improve patient outcomes, reduce healthcare costs, and enhance efficiency of healthcare delivery [5, 6].

In hospitals with medically complex patients and fiscal constraints, clinicians face high workloads with limited capacity, requiring them to prioritise care for those most at risk in a timely manner [7, 8]. A risk‐based approach can assist clinicians to focus their time and expertise on patients at greatest risk of medication harm without detracting from human judgement as to what care should be given [8]. While there is extensive literature on the development of RPTs [9], evidence of their successful clinical adoption and demonstrated impact on care outcomes specifically in the context of medication harm is limited [10]. Unlike many existing digital RPTs, medication harm RPTs are designed to support medication management decisions and prioritise patients for medication review [11, 12]. Their implementation may require integration into prescribing and medication review workflows, which can create distinct implementation challenges. Additionally, evidence suggests that medication error patterns are influenced by both patient characteristics and system‐level factors such as clinical setting, workflow, and medication complexity, further highlighting the importance of context in RPT implementation [13].

International literature has identified key considerations for implementing RPTs [14, 15, 16]. The prospective PRIME study (prospective study to develop a model to stratify the risk of medication‐related harm in hospitalised elderly patients) developed and internally validated a RPT for predicting medication harm following hospital discharge [17]. A subsequent qualitative study explored hospital pharmacists' views on the implementation and clinical use of the PRIME RPT [18]. This study provided valuable insights into pharmacists' perceptions of the tool, including potential enablers and barriers to its integration into clinical practice [18]. However, differences in health systems and user contexts highlight the importance of understanding local perspectives if RPTs are to be successfully integrated into Australian hospitals and improve patient outcomes while avoiding unintended consequences [19].

While general RPTs predict outcomes such as clinical deterioration or readmission, medication harm RPTs specifically predict the risk of harm related to medication use, such as adverse drug events or risks from polypharmacy and frailty [12, 18, 20]. In a preceding study, we implemented and evaluated the AIME‐Frail RPT to support pharmacists in identifying hospitalised patients at high risk of medication harm [12]. While the RPT demonstrated potential, its clinical impact was limited by suboptimal use of the tool among pharmacists [12]. This highlighted the need to better understand the factors influencing adoption and sustained use of digital RPTs in practice. In response, this qualitative study was conducted to explore the perspectives of clinicians and informaticians on the requirements, barriers, and facilitators for successful implementation of medication harm RPTs in hospital settings.

## 2 Study Aim and Research Questions

This study aimed to identify the professional and institutional requirements, and factors influencing the uptake of a digital RPT for medication harm. To address this aim, four key questions were explored with participants: 1) What are clinicians' perspectives on the use of digital RPTs for medication harm? 2) How can these tools be implemented in practice to assist clinicians with prioritising patients for medication review and management? 3) What specific challenges hinder the effective use of digital medication harm RPTs in practice? 4) What resources and supports are required from informaticians to design and implement such tools in the hospital setting?

## 3 Methods

### 3.1 Study Design

This was a qualitative study in which semi‐structured interviews were conducted with local doctors, pharmacists, and health informaticians using interview guides developed for each stakeholder group. The interview guides were piloted with two pharmacists to ensure the clarity and relevance of the research questions and to help researchers refine their interviewing approach. Minor adjustments were made based on feedback from the pilot. Data collected during the pilot interviews were not included in the final analysis. The study design was based on the Integrated Promoting Action on Research Implementation in Health Services (i‐PARIHS) framework [21], and results were reported according to the Consolidated Criteria for Reporting Qualitative Research (COREQ) statement [22].

### 3.2 Study Setting

This study took place at three digital tertiary hospitals within Queensland's Metro South Hospital and Health Service (MSH): Princess Alexandra Hospital (PAH), Queen Elizabeth II Jubilee Hospital (QE2), and Logan Hospital (LH). Hospitals under MSH operate within a digitally mature health service and use a state‐wide integrated electronic medical record (EMR) system [23]. The EMR included functionality such as computerised physician order entry, clinical documentation, and medication management [23]. Clinical decision support system features were embedded within the EMR and included early warning systems for clinical deterioration and medication alerts to support prescribing and clinical decision‐making [23].

MSH has developed a roadmap for digital health that emphasises digital innovation in care delivery and a 2023–2028 Digital Health Strategy that prioritises the use of predictive analytics [24]. MSH's transition to a fully digital health service has been associated with improvements in patient outcomes, clinician productivity, and operational efficiency [24]. The environment within this health system reflects digital maturity, which may have shaped participant expectations around digital risk prediction tools.

### 3.3 Risk Prediction Tool for Medication Harm

The study explored the perspectives of clinicians and informaticians on the use and implementation of digital RPTs for medication harm. AIME‐Frail was presented as an example of a medication harm RPT to facilitate discussion regarding implementation, usability, and clinical integration. The study did not aim to evaluate the AIME‐Frail tool itself.

The AIME‐Frail RPT was developed from routinely collected hospital data and contains predictors such as high‐risk medications, frailty, and length of stay (see supplementary file A) [12]. Each risk factor contributes to the overall risk score, with higher scores indicating a greater risk of harm. Clinicians were encouraged to prioritise patients for medication review and management from the highest to lowest risk based on the model's score [12]. Before the discussion, participants were verbally introduced to the AIME‐Frail RPT, including its predictors, risk scoring approach, and intended use in clinical practice.

### 3.4 Participants

Participants were recruited from PAH, QE2, and LH using a combination of purposive and snowball sampling to maximise the number of participants. Purposive sampling was used to ensure participants were in professional roles and had the expertise required to address the study's aims. Snowball sampling was used to broaden recruitment through participant referrals within existing professional networks to ensure all participants relevant to the research had the opportunity to participate. The study aimed to enrol up to 27 participants, comprising approximately nine individuals from each of the three stakeholder groups. This target was informed by a recent systematic review, which concluded that between nine and 17 interviews were typically sufficient to achieve data saturation in a relatively homogenous study population [25].

Recruitment strategies included sharing of study information at departmental meetings, disseminating email notices, and sending investigator messages to individuals. Potentially eligible participants were invited to complete a short survey indicating their availability and interest in the study.

Inclusion criteria for doctors and pharmacists were that they had to be working in direct patient care roles, with responsibilities involving prescribing, medication review, or clinical decision‐making, to ensure familiarity with the operational demands of inpatient care and the needs of frontline healthcare workers. They were also required to have a minimum of 6 months of hospital inpatient experience to ensure adequate exposure to local clinical workflows. Health informaticians were eligible if they had prior involvement in designing, implementing, or integrating digital health tools for clinical use in Australian hospitals, and routinely collaborated with clinicians in active informatics roles, such as data scientists, data analysts, informatics consultants, or informatics officers. Prior experience with RPTs was not required, as the study focused on general perspectives relevant to implementation.

### 3.5 Data Collection

Written consent was obtained from all participants prior to the interview by way of electronic signatures. Interviews were conducted over a 1‐month period from February to March 2025 and were limited to 30 minutes based on participant preference. Participants' demographic information pertaining to professional roles and years of experience in the hospital was requested prior to or during the interview. As the RPT had not been implemented at the time of interviews, participants had no preconceived views of its clinical use. The researchers did not hold supervisory roles over any participants, although some participants were known to them. Participation was entirely voluntary, and participants were informed they could skip questions or withdraw at any time.

All interviews were conducted either face‐to‐face or via Microsoft Teams, with participants interviewed from their workplace during regular working hours. Interviews were conducted by hospital pharmacists: JL (BPharm Hons) was a PhD candidate at the time of the study, and NF is a senior clinician researcher. Both have experience in developing RPTs such as AIME‐Frail and have extensive experience in qualitative research, the use of frameworks such as i‐PARIHS, and thematic analysis. Interviews were recorded using the platform's visual and audio recording function before being transcribed verbatim. Initial transcripts were all checked for accuracy and participant identifying information was removed to maintain confidentiality. Interview content was discussed only within the research team. All recorded interviews, transcripts, and related study documents were securely stored in the University of Queensland Research Data Manager.

The interview aimed to elicit the attitudes, knowledge, roles, and motivations of clinicians and informatics staff who may engage with the RPT. The interview guide used for these semi‐structured interviews was structured according to the i‐PARIHS framework and chosen because of its comprehensive and context‐sensitive approach to technology implementation [21]. Digital hospitals represent complex, dynamic environments where the success of new technologies like predictive models depends not only on the innovation itself but also on how well it aligns with the needs of healthcare professionals (recipients), the organisational and technological infrastructure (context), and the strategies used to support adoption (facilitation). i‐PARIHS provides a structured and adaptable framework for examining the various factors influencing implementation, making it appropriate for identifying barriers and enablers in clinical settings [21].

As described by Harvey et al. [21], the i‐PARIHS framework consists of four main constructs and associated subconstructs. In the context of this study, innovation refers to how the digital RPT is perceived in terms of its clarity, usefulness, credibility, and fit with clinical values and workflows, as well as its potential to improve on existing practice [21]. Recipients encompassed the attitudes, knowledge, roles, and motivations of clinicians and informatics staff who may engage with the RPT [21]. Context was defined as the organisational and local setting in which the tool will be implemented, including digital infrastructure, workflows, and leadership support [21]. Facilitation related to the processes, resources, and supports in place to enable implementation of RPTs into clinical practice [21].

### 3.6 Data Analysis

Inductive thematic analysis, as described by Braun and Clarke, was conducted to identify emergent themes and patterns from the data [26]. Manual coding was undertaken in Microsoft Word to capture recurring ideas and patterns, which were subsequently organised into themes. Coding involved iterative reading and review of interview transcripts as the study progressed.

The first reviewer (JL) coded all the transcripts, while a second reviewer (NF) independently coded six transcripts (27%), which were purposefully selected based on their richness of content and representation across the three stakeholder groups. These six transcripts were purposefully selected because they contained detailed participant responses, represented all three stakeholder groups, and provided in‐depth insight across a range of relevant themes, making them suitable for cross‐checking. Any inconsistencies between the two reviewers were resolved through consensus discussions. Once all transcripts were coded, the research team reviewed the codes and themes collectively and undertook an iterative process of refinement and validation of the findings.

Coding and initial themes were generated inductively from the interview data and then deductively mapped onto the i‐PARIHS framework. Additional themes and subthemes were identified, particularly where the framework highlighted aspects of implementation that were not initially captured through inductive analysis, with some themes aligned with multiple domains. The findings from the thematic analysis were then contextualised through reference to similar studies.

### 3.7 Methodological Rigour

In qualitative research, methodological rigour depends on key attributes such as credibility, transferability, dependability, and confirmability [27]. Credibility was supported by the investigators' hospital experience, expertise in medication safety and skills in the design and impact evaluation of the AIME‐Frail RPT. Additionally, the use of both inductive and deductive thematic analysis ensured that the themes were grounded in participant perspectives and informed by an established implementation framework in healthcare.

Transferability was achieved through the inclusion of three different stakeholder groups from three digital hospitals within MSH. This allowed the study to capture varied perspectives across different hospital contexts within a large integrated health system.

Dependability was ensured through regular peer debriefing sessions between JL and NF, where data collection processes, emerging codes, and challenges were discussed to promote consistent data interpretation. MB also assisted in resolving any coding disagreements that arose during these sessions. Additionally, a second reviewer (NF) independently coded a subset (27%) of all transcripts to identify discrepancies and support robust analysis.

Confirmability was maintained through ongoing reflexive team discussions to verify the authenticity of the coding and ensure the analysis remained accurate to the participants' perspectives and free of bias. The research team, consisting of a doctor, three pharmacists, and an informatician, verified the final coding to ensure a thorough and objective review of the analysis.

## 4 Results

Twenty‐two interviews were conducted involving six doctors, 11 pharmacists, and five informaticians. There was overlap among participants, as three pharmacists had transitioned into informatics roles, while others possessed both clinical and informatics expertise. Thematic saturation was achieved after 22 interviews, as determined by the repetition of themes and concepts across interviews and stakeholder groups, with additional data failing to yield further insights relevant to the research questions.

### 4.1 Participant Characteristics

Participant characteristics are described in Table 1. Most were less than 50 years of age, were pharmacists and male, had between 2‐ and 20‐years' experience, and worked at PAH.

**Table 1:** Participant Characteristics.

Characteristics	Options	Number (%)
Age of participants	20–29 years	3 (14)
	30–39 years	7 (32)
	40–49 years	6 (27)
	50–59 years	1 (5)
	60–69 years	5 (23)
Sex	Male	13 (59)
	Female	9 (41)
Years of experience	2–10 years	7 (32)
	11–20 years	10 (45)
	21–30 years	3 (14)
	31–40 years	2 (9)
Highest attained qualification	Bachelor's degree	8 (36)
	Graduate diploma or certificate	5 (23)
	Master's degree	3 (14)
	Doctorate degree	6 (27)
Occupation	Doctor	6 (27)
	Pharmacist	11 (50)
	Informaticians	5 (23)
Hospital sites	Princess Alexandra Hospital	17 (77)
	Logan hospital	3 (14)
	Queen Elizabeth Hospital	2 (9)

### 4.2 Themes

Five overarching themes encapsulating the professional and institutional requirements, and factors influencing the uptake of a digital RPT for medication harm were identified: Theme 1‐ Trust in the digital RPT, Theme 2‐ Desired features of digital RPTs, Theme 3‐ Suitability of digital RPTs in clinical settings, Theme 4‐ Organisational readiness and support for digital RPT implementation, and Theme 5‐ Enablers for digital RPT implementation. Fourteen subthemes were identified across these five major themes and were mapped to the relevant i‐PARIHS domains of innovation, recipients, context, and/or facilitation (see Table 2). Exemplar quotations were selected based on their representativeness of the themes and sub‐themes. Supporting quotations for all themes are provided in supplementary file B.

**Table 2:** Overview of Main Themes and Sub Themes and Their Mapping to i‐PARIHS Domains.

Main theme	Sub themes	i‐PARIHS domains
1. Trust in the digital risk prediction tool	Balancing trust in clinical judgement	Innovation, Recipients
	Adoption only when value is demonstrated	Recipients, Context
	Concerns about impact on clinical judgement	Innovation, Recipients
2. Desired features of digital risk prediction tools	Standardised responses to risk prediction tool outputs	Innovation, Recipients
	Minimising user burden through automation	Innovation
	Ability to override scores and provide feedback	Innovation, Recipients
3. Suitability of digital risk prediction tool in clinical settings	Risk thresholds in different clinical settings	Recipients, Context
	Integration into existing workflows	Innovation, Context
4. Organisational readiness and support for digital risk prediction tool implementation	Establish ownership and responsibility for oversight	Recipients, Context
	Leadership as a driver of stakeholder involvement and engagement	Context, Facilitation
	Ongoing audits and monitoring for quality assurance	Context
	Data quality and system interoperability challenges	Recipients, Context
5. Enablers for digital risk prediction tool implementation	Comprehensive feedback mechanisms for continuous improvement	Context, Facilitation
	Planning for adequate resources	Context

## 5 Theme 1: Trust in the Digital Risk Prediction Tool

### 5.1 Balancing Trust in Clinical Judgement

Clinicians reported that RPTs can be useful for triaging patients, provided they were evidence‐based and accurate. They emphasised that the tool should complement, rather than replace clinical expertise. Transparency in how risk scores are generated was seen as critical for fostering trust [28]. Informaticians also highlighted concerns about machine learning (ML) models as “black boxes”, reducing clinician confidence in outputs. This aligned with a systematic review by Rosenback et al. [29] which identified a lack of transparency in ML models as a barrier to clinician trust.

### 5.2 Adoption Only When Value Is Demonstrated

Participants reported that clinicians were generally willing to adopt digital RPTs when they demonstrated benefits for care or workflow efficiency. Clinician hesitation has been commonly reported in the literature, particularly during the introduction of new digital tools [30]. Our participants expressed that prior experience with digital systems facilitated adoption, while clinicians with limited experience using RPTs remained willing to use them when practical benefits were evident.

### 5.3 Concerns About Impact on Clinical Judgement

Clinicians expressed concerns that over‐reliance on RPTs could negatively affect clinical behaviour. They reported that excessive dependence may reduce autonomy and critical thinking. Participants' concerns were further supported by literature highlighting that digital tool overreliance may hinder the development of clinical skills among inexperienced clinicians, potentially limiting critical thinking and problem‐solving abilities necessary for independent practice [31, 32].

## 6 Theme 2: Desired Features of Digital Risk Prediction Tools

### 6.1 Standardised Responses to Risk Prediction Tool Outputs

Clinicians reported that clear and standardised protocols were essential for guiding decisions. Scoring systems, such as the Queensland Adult Deterioration Detection System (Q‐ADDS) [33], were cited as effective examples to promote consistent responses across teams. Participants highlighted that predefined actions linked to specific risk scores help reduce uncertainty and support practice consistency.

### 6.2 Minimising User Burden Through Automation

Participants reported a preference for digital RPTs that operate automatically with minimal manual input. Automation was seen as important for ensuring convenience, time efficiency, and integration into busy clinical workflows [34, 35]. Tools that require additional steps or manual data entry were considered burdensome and less likely to be used consistently. This aligned with existing literature highlighting that administrative burden can cause clinician stress, which may negatively affect the uptake and sustained use of new tools [36].

### 6.3 Ability to Override Scores and Provide Feedback

Participants reported that digital RPTs should allow clinicians to override risk scores when necessary, ensuring that clinical judgement remains central to decision‐making. They also highlighted the importance of embedding feedback mechanisms to capture user insights and support continuous improvement. Frequent overrides were seen as indicators of potential misalignment between the tool and actual clinical practice. Similarly, this aligned with existing literature indicating that overrides should be monitored and refined to improve tool relevance [37].

## 7 Theme 3: Suitability of Digital Risk Prediction Tool in Clinical Settings

### 7.1 Risk Thresholds in Different Clinical Settings

Clinicians reported that digital RPTs should balance sensitivity and specificity according to the needs of each clinical specialty. Appropriate thresholds were seen as important for enabling clinicians to focus on patients at the highest risk while minimising false positives and unnecessary alerts, ensuring the tool supports actionable decision‐making in practice. This aligned with findings from patient prioritisation of pharmacy services in the United Kingdom, where prioritisation tools sometimes lacked sensitivity across certain specialties, highlighting the importance of tailoring risk thresholds to specific clinical contexts [8].

### 7.2 Integration Into Existing Workflows

Participants reported that digital RPTs are less likely to be used if they are not integrated into existing clinical workflows and the EMR. Limited visibility, accessibility, and contextual relevance were seen as barriers to adoption. The tool was expected to prioritise patients in a manner that aligns with existing clinical workflows without introducing additional steps that could disrupt or delay care. While some workflow disruption is expected during implementation, aligning with existing natural workflows is essential to minimise interference and enhance user engagement with new tools [38].

## 8 Theme 4: Organisational Readiness and Support for Digital Risk Prediction Tool Implementation

### 8.1 Establish Ownership and Responsibility for Oversight

Participants reported that clear roles and responsibilities are essential for implementation, maintenance, and oversight of digital RPTs. They highlighted the importance of convening a multidisciplinary working group and assigning leadership roles to ensure the tool's sustainability and responsiveness to evolving clinical needs. Marwaha et al. [39] emphasised that clear ownership and sustained oversight are key to long‐term integration of digital health tools.

### 8.2 Leadership As a Driver of Stakeholder Involvement and Engagement

Participants reported that senior leadership support is critical for adoption as it boosts frontline staff confidence, secures necessary resources, and facilitates integration of the digital RPTs into the clinical workflows. They highlighted the value of appointing unit champions or superusers to provide training, troubleshooting, and peer‐to‐peer support. Engaging diverse stakeholders, including clinicians, informaticians, and hospital leadership, early in the process was highlighted as important to secure departmental representation. Similarly, the systematic review by Ingebrigtsen et al. [40] reported that institutional leaders with clinical and informatics expertise are the main drivers of staff engagement and collaboration, supporting successful implementation.

### 8.3 Ongoing Audits and Monitoring for Quality Assurance

Participants reported that ongoing audits and monitoring are essential to maintain the accuracy, timeliness, and trustworthiness of digital RPTs after implementation. Regular monitoring was seen as necessary to ensure the system continued to provide reliable information and support safe clinical decision‐making. In a health policy review, it was highlighted that monitoring digital tools over time is necessary to ensure they work as intended, particularly adaptive ML tools [41].

### 8.4 Data Quality and System Interoperability Challenges

Participants reported that interoperability issues can hinder effective implementation of digital RPTs, particularly those using ML models. Data extracted from EMRs are often incomplete, inconsistent, unstructured, or in incompatible formats, affecting the accuracy and usability of ML‐driven predictions [42]. Participants noted that data are rarely available in the right form for direct use in ML models, and that extensive data transformation and standardisation are often required to ensure compatibility with the model's processing requirements. Limited availability of key data at critical points, such as admission, was also identified as a barrier. Therefore, there is a need for preprocessing to optimise data quality, especially before ML model deployment [42].

## 9 Theme 5: Enablers for Digital Risk Prediction Tool Implementation

### 9.1 Comprehensive Feedback Mechanisms for Continuous Improvement

Participants reported that both pre‐ and post‐implementation feedback mechanisms are essential for the successful integration of digital RPTs. Ongoing feedback was seen as critical for identifying emerging issues, adapting the tool, and maintaining trust. Clinicians also highlighted the importance of gathering input from diverse specialties and levels of seniority to ensure the tool meets the needs of all stakeholders. Similarly, Romero‐Brufau et al. [43] reported that gathering feedback before and after implementation was necessary for their digital tool to fit within clinical workflows and promote sustained use.

### 9.2 Planning for Adequate Resources

Participants reported that successful use of digital RPTs depends on sufficient staffing, resources, and institutional support. Additional personnel were particularly important during the initial rollout to assist clinicians who were unfamiliar with the tool. Additionally, accessible training, ongoing technical support, and secured funding were important to maintain clinician engagement, build confidence, and enable scaling of the tool across clinical settings. This aligned with guidance from Marwaha et al. [39], who emphasised the importance of planning for adequate training, IT integration, information security, human capital investment, and adaptation of existing real‐world and EHR workflows to support effective digital tool implementation.

## 10 Discussion

### 10.1 Main Findings

This study provides insights into the views of clinicians and health informaticians on implementing digital RPTs for medication harm in hospitals. We identified a total of five themes and fourteen subthemes related to the implementation of medication harm RPTs in hospital settings. Themes highlighted that successful adoption depends not only on the tool's predictive performance and usability but also on clinician trust, acceptance, and alignment with clinical workflows and organisational processes. Participants emphasised the importance of designated ownership, robust governance, and ongoing quality assurance to support sustainable use. The design features that minimise user burden, such as automation, integration with existing systems, and options for clinician override and feedback, were necessary for uptake. Without these, issues like poor system integration, data quality issues, workflow misalignment, and unaddressed user feedback can undermine trust and limit adoption. These findings highlight the need for co‐designed, context‐specific strategies that address technical, human, and organisational factors to embed digital RPTs effectively in hospital care.

### 10.2 Comparison With Existing Literature

#### 10.2.1 Trust and Professional Autonomy in Using Risk Prediction Tools

While many participants indicated openness to adopting a novel RPT in their clinical practice, most had limited or no prior experience using such digital tools. Instead of history‐based trust which comes from direct experience, the trust that they referred to stemmed from a general willingness to place confidence in technology [44]. The basis of participants' trust was in relation to prior digital implementations, such as the transition from paper‐based charting to EMRs. This suggests that future digital RPT implementation should focus on fostering dispositional trust through early clinician engagement during RPT development [45, 46], while addressing scepticism from past digital implementations.

Trust in RPT outputs depended on perceived accuracy and alignment with clinical evidence, which strongly influence adoption [47]. Our findings support previous research showing digital RPTs are most effective when they complement, not replace clinical judgement [48]. Participants consistently emphasised the importance of retaining professional autonomy and concerns about over‐reliance on tools that override their clinical judgement, especially when tool reliability is unclear. This aligns with literature describing the potential for deskilling and loss of professional autonomy associated with dependence on automated predictions [49].

#### 10.2.2 Standardisation of Actions in Response to Tool Predictions

Some participants expressed that digital RPTs should be integrated with existing clinical protocols and pathways to standardise clinical actions taken across teams in response to tool predictions. This aligned with literature that described how such approaches reduce variability and improve care consistency [50]. Participants also believed that RPTs could assist in directing resources and staff time toward higher‐risk patients, which may help manage workloads and reduce burnout. This was consistent with evidence showing that digital tools improve workforce distribution and service delivery [51, 52, 53]. Additionally, demonstrating clinical benefits through better patient prioritisation or harm reduction may help gain clinician support for the implementation of RPTs in clinical practice [54].

#### 10.2.3 Performance Threshold Preferences in Different Settings

Participants highlighted that preferences for sensitivity and specificity thresholds varied depending on clinical context and clinician role. This concurs with prior research showing that the optimal trade‐off between sensitivity and specificity is context‐dependent [55]. Our participants expressed concern about excessive false positives overwhelming clinical workflows, especially in high acuity wards. Conversely, excessive false negatives may lead to missed clinical reviews or interventions for patients who are actually at high risk of harm. Therefore, RPTs should allow configurable thresholds tailored to clinical context and workforce capacity across hospital settings.

#### 10.2.4 Usability and Design Features to Reduce Burden

Participants highlighted the importance of usability, describing features such as automation, EMR integration, intuitive dashboards, and options to override or provide feedback as necessary for adoption. These preferences are consistent with broader literature identifying ease of use, workflow integration, and clinician control as key to digital tool uptake [56]. Participants were particularly concerned about alert fatigue and tools that disrupted rather than supported their clinical work, as emphasised by others [57]. The need for override options and feedback channels supports iterative refinement guided by clinician input [58].

#### 10.2.5 Organisational Readiness and Leadership Support

Participants described organisational readiness, which includes leadership support, designated ownership, and regular review processes, as important for successful RPT implementation. These findings are consistent with implementation science literature highlighting the need for strong leadership, governance clarity, and continuous quality monitoring to sustain digital tool use [59, 60]. Our participants also raised concerns that unclear responsibility for tool maintenance and optimisation would reduce long‐term engagement, and this has been noted in guidance proposed by other investigators [39]. For sustainable use, implementation of RPTs should be leadership‐driven and necessary roles should be established that are accountable for monitoring and updating tool performance.

#### 10.2.6 Facilitators of Implementation

Participants identified continuous feedback mechanisms and planning for adequate resources as facilitators of RPT implementation. Continuous feedback was considered essential for trust and clinical relevance because it enables iterative refinement. This aligns with the need for feedback loops to encourage clinician engagement and adoption [61, 62]. Additionally, pre‐implementation resource planning, including investment in infrastructure, adequate staffing, training and technical support, is necessary to ensure successful integration [63].

### 10.3 Additional Considerations From Existing Research

As with any intervention that impacts clinical decision‐making, it is important to assess potential equity and bias implications prior to RPT implementation. When RPTs lack transparency, clinicians, as the end‐users, may struggle to reconcile the RPT‐generated recommendations with clinical guidelines and patient preferences, which can lead to ethical dilemmas [64, 65]. Although RPTs can support more efficient resource allocation in resource‐constrained healthcare systems, their use should be balanced against ethical obligations to uphold patient rights and provide equitable access to care [65]. For example, prioritising patients at higher risk of harm for interventions based solely on RPT recommendations may unintentionally divert resources away from patients with lower risk who still require care [66]. This may compromise fairness in a universal health system where all patients should be entitled to the same standard of care regardless of their risk classification [66, 67]. Future studies need to investigate ways to balance efficiency gains from the use of RPTs with the obligation to maintain equitable care for all.

## 11 Practical Implementation Framework for Medication Harm Risk Prediction Tools

Based on the findings of this study, we developed a practical framework comprising four interconnected domains to support the successful implementation of medication harm RPTs.

### 11.1 Pre‐Implementation

#### 11.1.1 Prepare the Organisation

Implementation should begin with early stakeholder engagement, leadership support, and clear governance structures. Establishing ownership and accountability for oversight of the RPT, while ensuring adequate resourcing, staffing, and technical infrastructure, may promote organisational readiness and long‐term sustainability.

#### 11.1.2 Design for Practice

RPTs should be designed to integrate with existing workflows and information systems while minimising additional workload. Automation, interoperability, context‐specific risk thresholds, and clearly defined responses to risk predictions may improve usability and clinical relevance.

### 11.2 Implementation

#### 11.2.1 Support Adoption

Building clinician trust is critical for adoption. This may be achieved by demonstrating the value and performance of the RPT, providing appropriate training and support, and ensuring that the tool complements rather than replaces clinical judgement. Features allowing clinicians to override risk scores and provide feedback may further enhance acceptance.

### 11.3 Post‐Implementation

#### 11.3.1 Sustain and Optimise

Ongoing audit, monitoring, and review processes are required to ensure continued effectiveness. Mechanisms for user feedback, monitoring data quality, and refining the RPT over time may support continuous improvement and sustained use.

## 12 Strengths and Limitations

A strength of this study was the use of interviews to collect rich, in‐depth contextual data about the perceptions of RPTs from experienced stakeholders with diverse clinical and informatics roles in different hospitals. The semi‐structured interview format provided flexibility, allowing the interviewer to adapt questions based on participants' responses and enabling participants to share detailed accounts of their professional views, perceived barriers, and suggestions for improvement in ways that fully structured interviews could not facilitate. Another strength was the use of the i‐PARIHS framework to inform the design and analysis of the study. Applying this framework supported a more systematic and comprehensive understanding of implementation factors that might have been overlooked using an entirely inductive approach. It ensured all contextual, individual, and process‐related influences on RPT adoption were considered.

There were some limitations. Participants volunteered to take part in the study, which may have led to selection bias, with clinicians and informaticians who have a strong interest in digital RPTs being more likely to participate. Findings could be skewed towards the perspectives of stakeholders who are already advocates for digital health.

Another limitation is that the clinical specialty or area of practice of participating clinicians was not captured. As medication‐related risks and clinical decision‐making may vary across specialties, this may limit interpretation of the findings in relation to specific clinical contexts and types of medication harm.

This study did not include nursing staff, as the digital RPT was primarily designed to assist pharmacists in identifying and prioritising patients at high risk of medication harm. Doctors were included to provide perspectives on how pharmacist prioritisation can impact clinical decision‐making. While nurses are not the primary end‐users, their perspectives could provide complementary insights into how pharmacist‐led prioritisation aligns with broader multidisciplinary workflows.

The absence of a working AIME‐Frail RPT prototype may have limited participants' ability to fully appreciate the tool's usability, workflow implications, and value in clinical practice. In this study, participants were provided with a verbal description of the tool before the interview, and therefore perceptions may differ following actual use. Several participants described uncertainty in providing feedback because they had not seen a functional or visual representation of the tool. Participants also suggested that interactive demonstrations or visual mock‐ups would have provided more practical feedback.

Despite variations in health system structures, funding models, and policies, the factors identified in this study resonate with those reported internationally and reinforce the potential relevance and transferability of our findings to other groups aiming to implement digital RPTs.

## 13 Conclusion

This study identified key factors influencing the successful adoption of digital RPTs for medication harm in hospital settings. Findings highlight the need to align tool design with clinical workflows, maintain clinician autonomy, and maximise usability across diverse roles. Successful integration requires technical interoperability, organisational readiness, leadership support, clear governance structures, ongoing audit and feedback, and adequate user training and support. Early stakeholder engagement throughout implementation was considered important to ensure relevance and usability. These findings informed a practical implementation framework encompassing organisational preparation, workflow‐centred design, adoption support, and ongoing optimisation to guide implementation of medication harm RPTs in hospital settings. Future research should evaluate tailored strategies across sites to assess impacts on workflow, patient safety, and cost‐effectiveness.

## Ethics Statement

This study was approved by Metro South Health Human Research Ethics Committee (HREC/2023/QMS/99235). All participants provided written informed consent prior to participation in this study.

## Conflicts of Interest

The authors declare no conflicts of interest.

## Supporting information


Supporting File

## Data Availability

The data that support the findings of this study are available on request from the corresponding author. The data are not publicly available due to privacy or ethical restrictions.
