# Supplementary material for: An Evaluation of Clinician and Informatician Perspectives on Medication Harm Risk Prediction Tools for Use in a Tertiary Hospital Setting
Source: J Eval Clin Pract. 2026 Sep 13;32(6):e70592. doi: 10.1111/jep.70592 (PMC13571773; doi:10.1111/jep.70592)
Supplement: Supplementary file 1 [file JEP-32-0-s001.docx]

# Supplementary file A

**AIME-Frail Model**

| Variables | OR | 95% CIs | P-value | Points in Tool |
| --- | --- | --- | --- | --- |
| Intercept | 0.00 | 0.00 – 0.01 | <0.001 | N/A |
| 1. LoS > 14 days | 3.01 | 2.11 – 4.29 | 0.001 | +1 |
|  | Reference level: Not frail | | |  |
| 2. Frailty (CFS) |  |  |  |  |
| Moderate | 1.67 | 1.11 – 2.55 | 0.016 | + 1 |
| High | 3.92 | 2.41 – 6.42 | 0.001 | + 2 |
| 3. Anticoagulants | 2.23 | 1.30 – 4.11 | 0.006 | +1 |
| 4. Antipsychotics | 1.46 | 1.08 – 2.08 | 0.043 | +1 |
| 5.Immunosuppressants | 2.27 | 0.95 – 4.91 | 0.0493 | +1 |
| 6.Antiarrhythmics | 2.21 | 1.52 – 3.16 | 0.001 | +1 |
| 7. Opioid analgesics | 1.45 | 1.03 – 2.05 | 0.034 | +1 |
| 8. Insulin | 1.83 | 1.29 – 2.58 | 0.001 | +1 |
| 9. Antibiotics (All routes of administration) | 1.45 | 0.99 – 2.17 | 0.064 | +1 |

Key: CI is confidence intervals, CFS is Clinical Frailty Scale, LoS is length of stay, OR is odds ratio

# Supplementary file B

Trust in the digital risk prediction tool

| Number | Study ID | Quote |
| --- | --- | --- |
| Subtheme: Balancing trust in clinical judgement | | |
| 1 | DR3 | It’s not about replicating what clinicians already know. It's about offering new insights that might change their decision-making. As long as the output aligns with reality and provides meaningful, correct information, clinicians will likely trust and appreciate the tool more. |
| 2 | P1 | The tool should support and not override clinical judgment. |
| 3 | I1 | RPT needs to be evidence based… so that everyone feels comfortable. |
| 4 | I3 | Especially for machine learning models, it may be difficult to have an interpretation layer that explains how the model predicts… and because these solutions are new, people don’t really trust that well. |
| 5 | P3 | Clinicians need confidence that the RPT can assess risk as accurately as, or better than, they could do based on their own clinical judgement. |
| Subtheme: Adoption only when value is demonstrated | | |
| 1 | DR3 | Clinicians would be open to using a new tool if they can see that it adds value to their work and genuinely helps them |
| Subtheme: Concerns about impact on clinical judgement | | |
| 1 | DR5 | Clinicians might take the tool out of context and apply it incorrectly, lacking common sense in its application. Additionally, they may be overly reliant on it, which is why I am somewhat sceptical of many tools. |
| 2 | I2 | There is always a risk that we de-skill our clinicians, that we reduce their clinical autonomy. If our clinicians rely on tools to tell them when to do something, they will become poor clinicians. |

Desired features of digital risk prediction tools

| Number | Study ID | Quote |
| --- | --- | --- |
| Subtheme: Standardised responses to risk prediction tool outputs | | |
| 1 | DR1 | The organisation needs clear and consistent rules about what to do when a certain Q-ADDS score is reached. It means no one questions the response. For example, a Q-ADDS of eight typically justifies a Medical Emergency Team (MET) call. Lower scores might prompt discussion with the treating team or medical registrar. Some scores may simply indicate routine or more frequent observations. Because everyone understands the expected response, it removes uncertainty and helps standardise care. |
| Subtheme: Minimising user burden through automation | | |
| 1 | DR1 | The most convenient thing about it is that it is automated because many systems require someone to go out of their way to enter something or click something... going out of your way to do something is not conducive to being automated |
| 2 | DR2 | There’s not a lot of motivation for the team to do stuff that is quite labour-intensive. |
| Subtheme: Ability to override scores, and provide feedback | | |
| 1 | DR4 | We need the ability to override risk scores when necessary... however, if everybody is overriding something, then we need to reexamine it, as there may be a mismatch between the tool and what is expected theoretically or by expert knowledge… It’s also important that clinicians can provide feedback through the system so these issues can be identified and fixed over time. |

Suitability of digital risk prediction tool in clinical settings

| Number | Study ID | Quote |
| --- | --- | --- |
| Subtheme: Risk thresholds in difference clinical settings | | |
| 1 | DR1 | Some people could be worried about it, and I think there are some fair concerns, especially if the tool is not that sensitive or not specific or clinically inaccurate. |
| 2 | DR3 | When a tool is too sensitive, it often becomes too broad and identify so many cases that its output becomes difficult to act on. What would be more valuable is … determining how to intervene in a way that genuinely reduces readmissions. |
| Subtheme: Integration into existing workflows | | |
| 1 | DR5 | If the tool simply prioritises patients without any additional support or integration into their workflow, it might not be as well received… If it's not integrated directly within the EMR and easily accessible, people will likely struggle to use it as much. |
| 2 | DR2 | The priority of the medical officers is usually the patient being medically stable... from a workflow perspective, it's if they are being discharged |
| 3 | DR2 | We don't want to introduce a step that's going to be counterproductive because medical officers have their own priorities to assess patients. |
| 4 | DR3 | The tool shouldn’t require users to switch to an entirely separate system, like logging into a distant platform or printing out a list from a different computer. |

Organisational readiness and support for digital risk prediction tool implementation

| Number | Study ID | Quote |
| --- | --- | --- |
| Subtheme: Establish ownership and responsibility for oversight | | |
| 1 | DR2 | Someone has to take some leadership or ownership of the tool; that's often something that's easier said than done |
| 2 | I5 | A project working group should be convened early in the implementation process to oversee and ensure the reliability of the model |
| Subtheme: Leadership as a driver of stakeholder involvement and engagement | | |
| 1 | DR1 | At very least, having someone who is trusted, like our director involved in the project would at least ensure we feel represented. |
| 2 | P7 | Clinicians are going to embrace it wholeheartedly if you engage senior leadership at departments, you'll get a lot of support to implement this tool. |
| 3 | I1 | Clinical champions within whatever department you're trying to implement it in is always really useful... It works well when those champions are trained to then train other clinicians |
| 4 | I1 | You need to engage a good mix of people to give feedback on usability... especially the human factors team is really important because they have expertise in how the tool is visualised to the end users’ |
| 5 | P3 | Input from end users like pharmacists, doctors, and nurses, as well as support from data scientists and digital teams like E-Health to make sure the tool fits into the hospital’s existing systems. |
| Subtheme: Ongoing audits and monitoring for quality assurance | | |
| 1 | DR4 | You actually need an honest integrated digital system which communicates correct information, which is trustworthy and timely, and you've got to be able to audit. |
| Subtheme: Data Quality and system interoperability challenges | | |
| 1 | DR5 | The data in EMR is often incomplete or inconsistent... if data is inaccurate, the tool’s recommendations may not be reliable. |
| 2 | I3 | Especially for ML models, more focus needs to be put on the data transformation because the data in the system is never in the right form to develop these solutions |

Enablers for digital risk prediction tool implementation

| Number | Study ID | Quote |
| --- | --- | --- |
| Subtheme: Comprehensive feedback mechanisms for continuous improvement | | |
| 1 | DR1 | You need feedback before the tool is implemented, and I feel you do need follow up of the tool afterwards. |
| 2 | DR1 | The important thing about focus groups is making sure that we get representation from relevant specialties because what I think as a geriatrician and what I want might be very different to what I want as an acute general physician... I think that getting feedback from people at different levels involves having at least one person who perhaps can be a representative of registrars, at least a person who can represent junior doctors. |
| Subtheme: Planning for adequate resources | | |
| 1 | DR2 | There’s no use to predict patients are high risk or complex if you don’t have... manpower or resources for clinicians to look it up |
| 2 | DR4 | If your pharmacist is covering a few wards, then you need to consider if you could implement the tool. |
| 3 | DR1 | Extra staffing around the times that people were learning is really helpful... even if the person themselves were not tech literate, it meant you had an extra pair of hands to help if someone was struggling. |
| 4 | P4 | It would also be helpful to provide a step-by-step guide along with a designated support channel for technical issues. |
| 5 | P1 | You need grant funding... otherwise, you depend on people’s enthusiasm, which means scaling back the project. If clinicians are willing to volunteer their time... that would help address many resource concerns |
| 6 | DR3 | Large-scale implementation and evaluation are incredibly difficult if you haven’t done a solid pilot study first. But if that pilot is designed and executed well, it often provides enough insight to demonstrate that the outcomes and processes will scale effectively across the broader system. |
